# Benzene-1,3,5-triyl tris­(methane­sulfonate)

**DOI:** 10.1107/S1600536810006641

**Published:** 2010-03-03

**Authors:** Domingo Madrigal, Gerardo Aguirre, Berenice Vargas

**Affiliations:** a Centro de Graduados e Investigación del Instituto Tecnológico de Tijuana, Apdo. Postal 1166, 22500, Tijuana, B.C., Mexico

## Abstract

In the mol­ecule of the title compound, C_9_H_12_O_9_S_3_, the two methanesulfonate groups re located one above and one below the ring plane. The C—O—S angle range is 119.3 (2)–121.1 (2)°. This conformation is different from that of the benzene analog 1,2,5-tris­(*p*-toluene­sulfonate), which is a three-legged ‘table’ with all fragments of the *p*-toluene­sulfonate on top of the benzene ring.  In the crystal, the supra­molecular aggregation is completed by the presence of C—H⋯O hydrogen bonds.

## Related literature

For infrared spectroscopic studies related compounds, see: Grice *et al.* (2000 ▶); Yan & Yan (2001 ▶). For mass spectroscopy of related compounds, see: Chavez *et al.* (2003 ▶)**;** Olivas *et al.* (2008 ▶); Madrigal *et al.* (2006 ▶). For examples of O⋯O inter­actions, see: Raghavaiah *et al.* (2006 ▶), and for a comprehensive theoretical treatment, see: Ni *et al.* (2004 ▶). For a related structure, see:, see: Vembu *et al.* (2003 ▶). For the IR spectrum, see: Skoog *et al.* (1997 ▶).
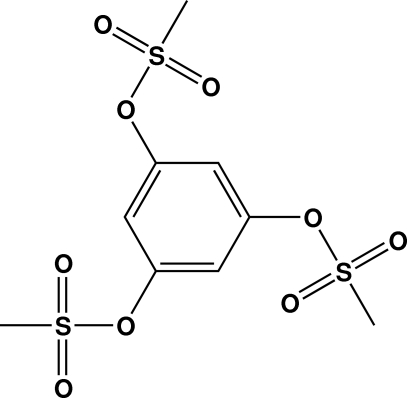


## Experimental

### 

#### Crystal data

C_9_H_12_O_9_S_3_
*M* *_r_* = 360.37
Monoclinic, 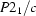

*a* = 8.7810 (5) Å
*b* = 17.0053 (9) Å
*c* = 9.7746 (7) Å
β = 100.595 (5)°
*V* = 1434.69 (15) Å^3^
*Z* = 4
Mo *K*α radiation
μ = 0.56 mm^−1^
*T* = 298 K
0.40 × 0.24 × 0.10 mm

#### Data collection

Bruker P4 diffractometer
Absorption correction: ψ scan (*XSCANS*; Siemens, 1996 ▶) *T*_min_ = 0.258, *T*_max_ = 0.310
4418 measured reflections
4176 independent reflections
2333 reflections with *I* > 2σ(*I*)
*R*_int_ = 0.048
3 standard reflections every 97 reflections  intensity decay: 4.3%

#### Refinement

*R*[*F* ^2^ > 2σ(*F* ^2^)] = 0.055
*wR*(*F* ^2^) = 0.177
*S* = 0.92
4176 reflections
190 parameters
H-atom parameters constrained
Δρ_max_ = 0.32 e Å^−3^
Δρ_min_ = −0.33 e Å^−3^

### 

Data collection: *XSCANS* (Siemens, 1996 ▶); cell refinement: *XSCANS*; data reduction: *XSCANS*; program(s) used to solve structure: *SHELXS97* (Sheldrick, 2008 ▶); program(s) used to refine structure: *SHELXL97* (Sheldrick, 2008 ▶); molecular graphics: *Mercury* (Macrae *et al.*, 2006 ▶); software used to prepare material for publication: *SHELXL97*.

## Supplementary Material

Crystal structure: contains datablocks I, global. DOI: 10.1107/S1600536810006641/bg2322sup1.cif

Structure factors: contains datablocks I. DOI: 10.1107/S1600536810006641/bg2322Isup2.hkl

Additional supplementary materials:  crystallographic information; 3D view; checkCIF report

## Figures and Tables

**Table 1:** Hydrogen-bond geometry (Å, °)

*D*—H⋯*A*	*D*—H	H⋯*A*	*D*⋯*A*	*D*—H⋯*A*
C2—H2*A*⋯O5^i^	0.93	2.51	3.392 (4)	159
C9—H9*D*⋯O4^ii^	0.96	2.32	3.259 (5)	166
C4—H4*A*⋯O6^iii^	0.93	2.52	3.444 (4)	172
C9—H9*C*⋯O8^iv^	0.96	2.55	3.312 (5)	136

Symmetry codes: (i) 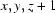; (ii) 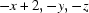; (iii) 
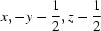
; (iv) 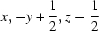.

**Fig. 1.**
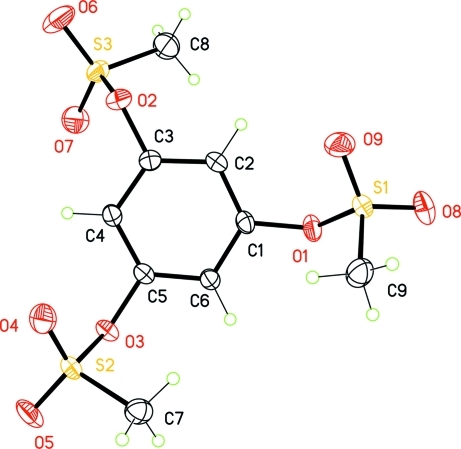
The title compound (I) with displacement ellipsoids drawn at a 30% probability level.

**Fig. 2.**
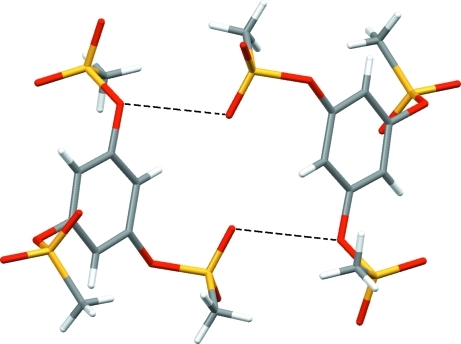
The molecules forming cyclic dimers. O—O bonds are indicated by broken lines.

## supplementary crystallographic information

### Comment

The synthesis of supported organic compounds and dendrimers on Merrifield resin with a mesyl group and a trihydroxybenzene core has been one of our objectives; however the analysis of the supported products on the solid state is only limited to infrared spectroscopy (Grice *et al.*, 2000; Yan *et al.*, 2001). In our previous work we have used mass spectroscopy to characterize intermediates and products (Chavez *et al.*, 2003; Olivas *et al.*, 2008;. Madrigal *et al., *2006). In this work, and as part of our ongoing research, we have synthesized benzene-1,3,5-triyl trimethanesulfonate using 1,3,5 trihydroxybenzene. The product (I) is an intermediate in the synthesis of complex first , second and third generation dendrimers.

As shown in Fig. 1, the molecule shows two fragments of trimethanesulfonate above and one below the plane of the benzene ring, with angles C5—O3—S2 119.3 (2)° , C1—01—S1 121.1 (2)° and C3—02—S3 120.2 (2)°. This conformation is different from the one shown by the analogue benzene 1,2,5-tris(*p*-toluenesulfonate), where the conformation of the molecule is described as a three-legged table (all fragments of the *p*-toluenesulfonate lay on the top of the benzene ring) stabilized by intramolecular C—H···O and C— H···π (Vembu *et al.*, 2003).

In the crystal structure of (I), adjacent units are arranged like dimers via intermolecular C3—O2···(O9—S1)^i^, (3.035 Å, (i): 2-x,-y,1-z ) oxygen bond interactions (Fig 2). Similar interactions are described in the literature, e.g. the helical structure of sulfate anions formed by non-covalent O···O (2.9413 Å) contacts described in Raghavaiah *et al.* 2006, and it stresses the role of a flip-flop water chain in determining the helical arrangement of sulfate anions in the solid state. These non-covalent O···O interactions are well-established in the literature including their theoretical aspects (Ni *et al.* 2004). In addition, adjacent dimers are linked together *via* intermolecular C—H···O hydrogen bond interactions (table 2).

### Experimental

The synthesis of the title compound included reagents and solvents of reagent grade, which were used without further purification. In a round bottom flask of 10 ml provided with a magnetic stirrer, was placed 0.3 g (2.3 mmol) of 1,3,5-trihydroxybenzene and 3 ml of pyridine. The flask was immersed in an ice bath and 0.58 ml (7.6 mmol) of methanesulfonyl chloride was added dropwise. The mixture was stirred for one hour and stored in the refrigerator for 24 hours. The reaction mixture was poured on to cracked ice and the precipitate was washed with a cold solution 20% of HCl (3 x 5 ml) and cold water (3 x 5 ml). The solid obtained was dried under vacuum. The yield was of 49 % of melting point: 142 °C. IR(KBr): 3099, 3027, 1602, 1458, 1367, 1182, 1110 cm^-1 ^(Skoog, *et al.*, 1997). ^1^H NMR (CDCl_3_): δ 7.51 (s, 3H, CH), 3.49 (s, 9H, CH_3_). ^13^C NMR (CDCl_3_): δ 149.7(CO), 116.3(CH), 39.1(CH_3_).

Crystallization.

50 mg of benzene-1,3,5-triyl trimethanesulfonate compound was placed in a glass vial and 3 ml of dimethyl sulfoxide was added. The solution was allowed to stand at room temperature for seven days and the crystals formed were separated by filtration.

### Refinement

Refinement for H atoms was carried out using a riding model, with distances constrained to: 0.93 Å for aromatic CH, 0.98 Å for methine CH. Isotropic displacement parameters were fixed to Uĩso~(H)=1.2/1.5 U~eq~(carrier atom)

### Crystal data

**Table:**

C_9_H_12_O_9_S_3_	*F*(000) = 744
*M**_r_* = 360.37	*D*_x_ = 1.668 Mg m^−^^3^
Monoclinic, *P*2_1_/*c*	Mo *K*α radiation, λ = 0.71073 Å
Hall symbol: -P 2ybc	Cell parameters from 51 reflections
*a* = 8.7810 (5) Å	θ = 4.9–12.4°
*b* = 17.0053 (9) Å	µ = 0.56 mm^−^^1^
*c* = 9.7746 (7) Å	*T* = 298 K
β = 100.595 (5)°	Needle, colourless
*V* = 1434.69 (15) Å^3^	0.40 × 0.24 × 0.10 mm
*Z* = 4	

### Data collection

**Table:**

Bruker P4 diffractometer	2333 reflections with *I* > 2σ(*I*)
Radiation source: fine-focus sealed tube	*R*_int_ = 0.048
graphite	θ_max_ = 30.0°, θ_min_ = 2.4°
2θ/ω scans	*h* = 0→12
Absorption correction: ψ scan (*XSCANS*; Siemens, 1996)	*k* = 0→23
*T*_min_ = 0.258, *T*_max_ = 0.310	*l* = −13→13
4418 measured reflections	3 standard reflections every 97 reflections
4176 independent reflections	intensity decay: 4.3%

### Refinement

**Table:**

Refinement on *F*^2^	Primary atom site location: structure-invariant direct methods
Least-squares matrix: full	Secondary atom site location: difference Fourier map
*R*[*F*^2^ > 2σ(*F*^2^)] = 0.055	Hydrogen site location: inferred from neighbouring sites
*wR*(*F*^2^) = 0.177	H-atom parameters constrained
*S* = 0.92	*w* = 1/[σ^2^(*F*_o_^2^) + (0.1*P*)^2^] where *P* = (*F*_o_^2^ + 2*F*_c_^2^)/3
4176 reflections	(Δ/σ)_max_ < 0.001
190 parameters	Δρ_max_ = 0.32 e Å^−^^3^
0 restraints	Δρ_min_ = −0.33 e Å^−^^3^

### Special details

**Table:**

Geometry. All esds (except the esd in the dihedral angle between two l.s. planes) are estimated using the full covariance matrix. The cell esds are taken into account individually in the estimation of esds in distances, angles and torsion angles; correlations between esds in cell parameters are only used when they are defined by crystal symmetry. An approximate (isotropic) treatment of cell esds is used for estimating esds involving l.s. planes.
Refinement. Refinement of *F*^2^ against ALL reflections. The weighted *R*-factor wR and goodness of fit *S* are based on *F*^2^, conventional *R*-factors *R* are based on *F*, with *F* set to zero for negative *F*^2^. The threshold expression of *F*^2^ > σ(*F*^2^) is used only for calculating *R*-factors(gt) etc. and is not relevant to the choice of reflections for refinement. *R*-factors based on *F*^2^ are statistically about twice as large as those based on *F*, and *R*- factors based on ALL data will be even larger.

### Fractional atomic coordinates and isotropic or equivalent isotropic displacement parameters (Å^2^)

**Table:**

	*x*	*y*	*z*	*U*_iso_*/*U*_eq_	
S1	0.88376 (11)	0.16428 (6)	0.42609 (9)	0.0390 (2)	
S2	0.78601 (11)	0.00358 (6)	−0.20344 (8)	0.0376 (2)	
S3	0.60481 (12)	−0.19992 (6)	0.32307 (10)	0.0441 (3)	
O3	0.6500 (3)	0.00837 (15)	−0.1152 (2)	0.0362 (5)	
O2	0.7498 (3)	−0.14794 (15)	0.3008 (3)	0.0414 (6)	
O1	0.7281 (3)	0.13465 (16)	0.3276 (2)	0.0431 (6)	
C2	0.7397 (4)	−0.0073 (2)	0.3185 (3)	0.0342 (7)	
H2A	0.7576	−0.0108	0.4151	0.041*	
C6	0.6987 (4)	0.0718 (2)	0.1073 (3)	0.0322 (7)	
H6A	0.6893	0.1207	0.0640	0.039*	
C4	0.7008 (4)	−0.0705 (2)	0.0917 (3)	0.0322 (7)	
H4A	0.6932	−0.1159	0.0380	0.039*	
C5	0.6872 (4)	0.0031 (2)	0.0319 (3)	0.0307 (7)	
C3	0.7265 (4)	−0.0735 (2)	0.2358 (3)	0.0330 (7)	
C1	0.7251 (4)	0.0646 (2)	0.2512 (3)	0.0323 (7)	
O5	0.7038 (4)	−0.00487 (19)	−0.3418 (3)	0.0606 (9)	
O4	0.8956 (4)	−0.05374 (18)	−0.1437 (3)	0.0564 (8)	
C7	0.8698 (5)	0.0967 (2)	−0.1803 (4)	0.0463 (9)	
H7A	0.9541	0.1000	−0.2302	0.069*	
H7B	0.9079	0.1056	−0.0830	0.069*	
H7C	0.7936	0.1359	−0.2148	0.069*	
O9	0.9613 (4)	0.09894 (18)	0.4963 (3)	0.0619 (9)	
O8	0.8301 (4)	0.22633 (19)	0.5033 (3)	0.0602 (8)	
O7	0.4863 (4)	−0.19317 (19)	0.2031 (3)	0.0626 (8)	
O6	0.6727 (4)	−0.27378 (18)	0.3633 (4)	0.0759 (10)	
C9	0.9942 (5)	0.2029 (3)	0.3109 (5)	0.0559 (11)	
H9B	1.0906	0.2223	0.3622	0.084*	
H9C	0.9385	0.2452	0.2590	0.084*	
H9D	1.0143	0.1625	0.2479	0.084*	
C8	0.5441 (6)	−0.1548 (3)	0.4643 (5)	0.0608 (12)	
H8B	0.4558	−0.1823	0.4855	0.091*	
H8C	0.5165	−0.1012	0.4413	0.091*	
H8D	0.6267	−0.1562	0.5436	0.091*	

### Atomic displacement parameters (Å^2^)

**Table:**

	*U*^11^	*U*^22^	*U*^33^	*U*^12^	*U*^13^	*U*^23^
S1	0.0487 (5)	0.0386 (5)	0.0283 (4)	−0.0024 (4)	0.0034 (4)	−0.0045 (4)
S2	0.0461 (5)	0.0422 (5)	0.0263 (4)	0.0004 (4)	0.0117 (3)	−0.0044 (4)
S3	0.0552 (6)	0.0307 (5)	0.0477 (5)	−0.0008 (4)	0.0132 (4)	0.0047 (4)
O3	0.0369 (12)	0.0483 (15)	0.0229 (10)	−0.0022 (11)	0.0046 (9)	−0.0009 (10)
O2	0.0424 (14)	0.0375 (14)	0.0441 (14)	0.0047 (11)	0.0076 (11)	0.0099 (11)
O1	0.0468 (15)	0.0410 (14)	0.0376 (13)	0.0089 (12)	−0.0021 (11)	−0.0150 (11)
C2	0.0375 (17)	0.0410 (19)	0.0238 (14)	0.0003 (15)	0.0043 (12)	0.0003 (14)
C6	0.0343 (17)	0.0335 (17)	0.0283 (15)	0.0000 (14)	0.0042 (13)	−0.0020 (13)
C4	0.0338 (17)	0.0338 (17)	0.0296 (16)	−0.0035 (14)	0.0072 (13)	−0.0048 (13)
C5	0.0297 (15)	0.0382 (17)	0.0244 (14)	−0.0032 (14)	0.0056 (12)	−0.0015 (14)
C3	0.0316 (17)	0.0335 (17)	0.0341 (16)	0.0002 (14)	0.0062 (14)	0.0028 (14)
C1	0.0325 (17)	0.0363 (18)	0.0287 (15)	0.0047 (14)	0.0066 (13)	−0.0075 (14)
O5	0.076 (2)	0.081 (2)	0.0242 (12)	−0.0115 (18)	0.0093 (13)	−0.0115 (13)
O4	0.0625 (19)	0.0546 (18)	0.0561 (17)	0.0207 (15)	0.0218 (14)	−0.0017 (14)
C7	0.046 (2)	0.048 (2)	0.046 (2)	−0.0071 (18)	0.0088 (18)	0.0034 (18)
O9	0.069 (2)	0.0528 (18)	0.0534 (17)	−0.0063 (15)	−0.0173 (15)	0.0123 (14)
O8	0.069 (2)	0.066 (2)	0.0465 (16)	−0.0050 (16)	0.0130 (14)	−0.0271 (15)
O7	0.065 (2)	0.0585 (19)	0.0591 (18)	−0.0198 (16)	−0.0012 (16)	−0.0021 (15)
O6	0.095 (3)	0.0401 (17)	0.099 (3)	0.0165 (17)	0.036 (2)	0.0248 (17)
C9	0.071 (3)	0.044 (2)	0.059 (2)	−0.005 (2)	0.028 (2)	−0.002 (2)
C8	0.073 (3)	0.062 (3)	0.053 (2)	0.001 (2)	0.028 (2)	0.001 (2)

### Geometric parameters (Å, °)

**Table:**

S1—O9	1.413 (3)	C2—H2A	0.9300
S1—O8	1.427 (3)	C6—C5	1.375 (5)
S1—O1	1.601 (3)	C6—C1	1.389 (4)
S1—C9	1.744 (4)	C6—H6A	0.9300
S2—O4	1.418 (3)	C4—C5	1.377 (5)
S2—O5	1.418 (3)	C4—C3	1.385 (4)
S2—O3	1.598 (3)	C4—H4A	0.9300
S2—C7	1.744 (4)	C7—H7A	0.9600
S3—O6	1.415 (3)	C7—H7B	0.9600
S3—O7	1.422 (3)	C7—H7C	0.9600
S3—O2	1.598 (3)	C9—H9B	0.9600
S3—C8	1.745 (4)	C9—H9C	0.9600
O3—C5	1.417 (4)	C9—H9D	0.9600
O2—C3	1.414 (4)	C8—H8B	0.9600
O1—C1	1.404 (4)	C8—H8C	0.9600
C2—C3	1.379 (5)	C8—H8D	0.9600
C2—C1	1.383 (5)		
			
O9—S1—O8	120.1 (2)	C3—C4—H4A	121.6
O9—S1—O1	109.04 (16)	C6—C5—C4	123.5 (3)
O8—S1—O1	102.84 (16)	C6—C5—O3	118.1 (3)
O9—S1—C9	109.5 (2)	C4—C5—O3	118.3 (3)
O8—S1—C9	109.9 (2)	C2—C3—C4	123.1 (3)
O1—S1—C9	104.17 (19)	C2—C3—O2	118.5 (3)
O4—S2—O5	120.70 (19)	C4—C3—O2	118.2 (3)
O4—S2—O3	109.32 (16)	C2—C1—C6	122.9 (3)
O5—S2—O3	102.73 (16)	C2—C1—O1	120.4 (3)
O4—S2—C7	109.43 (19)	C6—C1—O1	116.6 (3)
O5—S2—C7	110.14 (19)	S2—C7—H7A	109.5
O3—S2—C7	102.88 (17)	S2—C7—H7B	109.5
O6—S3—O7	120.5 (2)	H7A—C7—H7B	109.5
O6—S3—O2	102.92 (19)	S2—C7—H7C	109.5
O7—S3—O2	108.86 (16)	H7A—C7—H7C	109.5
O6—S3—C8	110.1 (2)	H7B—C7—H7C	109.5
O7—S3—C8	109.5 (2)	S1—C9—H9B	109.5
O2—S3—C8	103.4 (2)	S1—C9—H9C	109.5
C5—O3—S2	119.3 (2)	H9B—C9—H9C	109.5
C3—O2—S3	120.2 (2)	S1—C9—H9D	109.5
C1—O1—S1	121.1 (2)	H9B—C9—H9D	109.5
C3—C2—C1	116.9 (3)	H9C—C9—H9D	109.5
C3—C2—H2A	121.5	S3—C8—H8B	109.5
C1—C2—H2A	121.5	S3—C8—H8C	109.5
C5—C6—C1	116.8 (3)	H8B—C8—H8C	109.5
C5—C6—H6A	121.6	S3—C8—H8D	109.5
C1—C6—H6A	121.6	H8B—C8—H8D	109.5
C5—C4—C3	116.8 (3)	H8C—C8—H8D	109.5
C5—C4—H4A	121.6		
			
O4—S2—O3—C5	39.6 (3)	S2—O3—C5—C4	−85.2 (3)
O5—S2—O3—C5	168.9 (3)	C1—C2—C3—C4	0.4 (5)
C7—S2—O3—C5	−76.7 (3)	C1—C2—C3—O2	176.3 (3)
O6—S3—O2—C3	168.7 (3)	C5—C4—C3—C2	−0.6 (5)
O7—S3—O2—C3	39.7 (3)	C5—C4—C3—O2	−176.4 (3)
C8—S3—O2—C3	−76.6 (3)	S3—O2—C3—C2	98.7 (3)
O9—S1—O1—C1	−40.3 (3)	S3—O2—C3—C4	−85.2 (3)
O8—S1—O1—C1	−168.7 (3)	C3—C2—C1—C6	−0.1 (5)
C9—S1—O1—C1	76.6 (3)	C3—C2—C1—O1	176.3 (3)
C1—C6—C5—C4	−0.2 (5)	C5—C6—C1—C2	0.0 (5)
C1—C6—C5—O3	175.9 (3)	C5—C6—C1—O1	−176.5 (3)
C3—C4—C5—C6	0.4 (5)	S1—O1—C1—C2	69.1 (4)
C3—C4—C5—O3	−175.6 (3)	S1—O1—C1—C6	−114.3 (3)
S2—O3—C5—C6	98.5 (3)		

### Hydrogen-bond geometry (Å, °)

**Table:**

*D*—H···*A*	*D*—H	H···*A*	*D*···*A*	*D*—H···*A*
C2—H2A···O5^i^	0.93	2.51	3.392 (4)	159
C9—H9D···O4^ii^	0.96	2.32	3.259 (5)	166
C4—H4A···O6^iii^	0.93	2.52	3.444 (4)	172
C9—H9C···O8^iv^	0.96	2.55	3.312 (5)	136

Symmetry codes: (i) *x*, *y*, *z*+1; (ii) −*x*+2, −*y*, −*z*; (iii) *x*, −*y*−1/2, *z*−1/2; (iv) *x*, −*y*+1/2, *z*−1/2.
